# Valence tautomerism in a cobalt–dioxolene complex containing an imidazolic ancillary ligand†‡

**DOI:** 10.1039/d3ra03235c

**Published:** 2023-07-04

**Authors:** Anderson Moledo Vicente Guedes, Leandro Sodré de Abreu, Igor Antunes Vogel Maldonado, William Silva Fernandes, Thiago Messias Cardozo, Rafael A. Allão Cassaro, Marciela Scarpellini, Giordano Poneti

**Affiliations:** a Instituto de Química, Universidade Federal do Rio de Janeiro Rio de Janeiro RJ 21941-909 Brazil giordano.poneti@unitus.it

## Abstract

This work reports the synthesis, structural, spectroscopic and magnetic investigation of two complexes, [Co(bmimapy)(3,5-DTBCat)]PF_6_·H_2_O (1) and [Co(bmimapy)(TCCat)]PF_6_·H_2_O (2), where bmimapy is an imidazolic tetradentate ancillary ligand and 3,5-DTBCat and TCCat are the 3,5-di-*tert*-butyl-catecholate and tetrachlorocatecholate anions, respectively. Their structures have been elucidated using single crystal X-ray diffraction, showing a pseudo-octahedral cobalt ion bound to a chelating dioxolene ligand and the ancillary bmimapy ligand in a folded conformation. Magnetometry displayed an entropy-driven, incomplete, Valence Tautomeric (VT) process for 1 in the 300–380 K temperature range, while 2 displayed a temperature independent, diamagnetic low-spin cobalt(iii)–catecholate charge distribution. This behaviour was interpreted on the basis of the cyclic voltammetric analysis, allowing the estimation of the free energy difference associated with the VT interconversion of +8 and +96 kJ mol^−1^ for 1 and 2, respectively. A DFT analysis of this free energy difference highlighted the ability of the methyl-imidazole pendant arm of bmimapy favouring the onset of the VT phenomenon. This work introduces the imidazolic bmimapy ligand to the scientific community working in the field of valence tautomerism, increasing the library of ancillary ligands to prepare temperature switchable molecular magnetic materials.

## Abstract 2

The imidazolic tetradentate ligand bmimapy is used to prepare a new valence tautomeric coordination compound.

## Graphical abstract

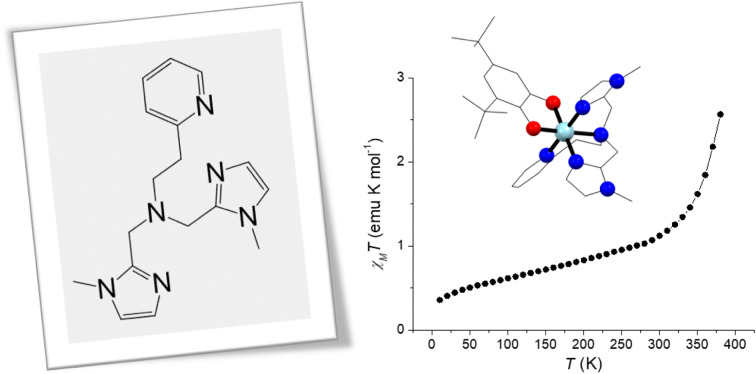

## Introduction

Molecular systems that can reversibly alter their physical properties under an external stimulus are investigated as potential new materials for different kinds of applications, including treatment^1^ or storage^2–10^ of data, sensors^11–13^ or mechanical actuators.^14^ Valence tautomeric materials indeed fit into this picture, featuring the possibility to reversibly trigger an intramolecular electron transfer between a metal ion and a non-innocent organic ligand upon heat, pressure, magnetic field or light stimuli,^15–23^ in the condensed phase,^24^ solution,^25^ nanoparticles, thin films^26^ and even as a self-assembled monolayer on a conductive surface.^27^ Despite having been observed for different transition metal ions,^19^ the majority of coordination compounds undergoing Valence Tautomerism (VT) contains a cobalt ion bound to one or two dioxolene (diox, *i.e.*: *ortho*-dihydroxo-benzene) ligands; in this case, the intramolecular electron transfer triggers a spin state change of the metal ion, thus defining two different electronic states for the system: low-spin Co(iii)Cat (Cat = dianion of the catecholate redox form of the dioxolene ligand – ls-Co^III^Cat) and high-spin Co(ii)SQ (SQ = monoanionic semiquinonate form of the dioxolene – hs-Co^II^SQ). Such a rearrangement of the intramolecular charge distribution leads to huge variations in the structural, spectroscopic and magnetic properties of the coordination compounds, thereby justifying their utilization in devices incorporating switchable active units.

The driving force of the VT equilibrium is the entropy gain of the system on passing from the ls-Co^III^Cat electronic isomer, with stronger bonds in the first coordination sphere and lower molar enthalpy, to the hs-Co^II^SQ one, featuring higher density of vibrational states, higher spin multiplicity, and therefore higher molar entropy.^24,28–30^ For the VT phenomenon to occur, the difference between the free energies of the two electromers involved in the process, ls-Co^III^Cat and hs-Co^II^SQ, must be close to the thermal energy at room temperature. Dei and co-workers related this quantity to the difference in the oxidation potential of the metal ion and the dioxolene ligand in the neutral [M(ii)(L)(Cat)] species, being L an ancillary, tetradentate ligand, completing the pseudo-octahedral coordination sphere of the metal ion.^28,31^ Moreover, they showed that chemical variation in the structure of the ancillary ligand can drastically alter the VT behaviour for the family of [Co(Me_*n*_tpa)(3,5-DTBCat)]^+^ complexes, where Me_*n*_tpa are derivatives of the tris-(2-pyridylmethyl)amine bearing methyl-pyridine pendant arms, substituted with *n* methyl groups in the *ortho* position (*n* = 0–3). In particular, for *n* = 0 and 1 a temperature independent ls-Co^III^Cat charge distribution was observed, while for *n* = 3 a hs-Co^II^SQ one was found, while the [Co(Me_2_tpa)(diox)]^+^ displayed VT in solution and in the solid state.^21,28,32,33^ A similar approach for the chemical control of charge distribution in cobalt : dioxolene complexes of formula [Co(Pz_*n*_Py_3−*n*_)(3,5-DTBCat)]^+^ was achieved by Yu and Li.^34^ In their work they employed, as ancillary ligand, the Pz_*n*_Py_3−*n*_ tripodal ligand, featuring a variable number of pyridine (Py) and dimethylated pyrazolyl (Pz) moieties connected to a central tertiary nitrogen atom through a methylene group. They showed that, for *n* = 1, a ls-Co^III^Cat charge distribution is the ground state in the 2–300 K temperature range, while for *n* = 2 and 3 a temperature independent hs-Co^II^SQ one is found, pointing out that the pyridine moiety is able to stabilize the ls-Co^III^ ion, while the methylated pyrazolyl groups favour the hs-Co^II^ one. In the case of [Co(diox)_2_(N–N)] complexes, the role of the diazine N–N ligand was investigated by Pierpont and coworkers, showing how the donating properties^35^ and the flexibility^36^ of the diazine ligand affect the VT transition temperature. More recently, Boskovic, Goerigk and co-workers found a linear correlation between the VT transition temperature and the energy of the LUMO of the diazine ligand in a remarkably large temperature interval (150 K) in similar systems.^37^ Boskovic and co-workers, later on, used the same electrochemical approach to rationalize the presence of multi-step transitions in cobalt complexes with a ditopic dioxolene ligand.^38^

**Chart 1:**
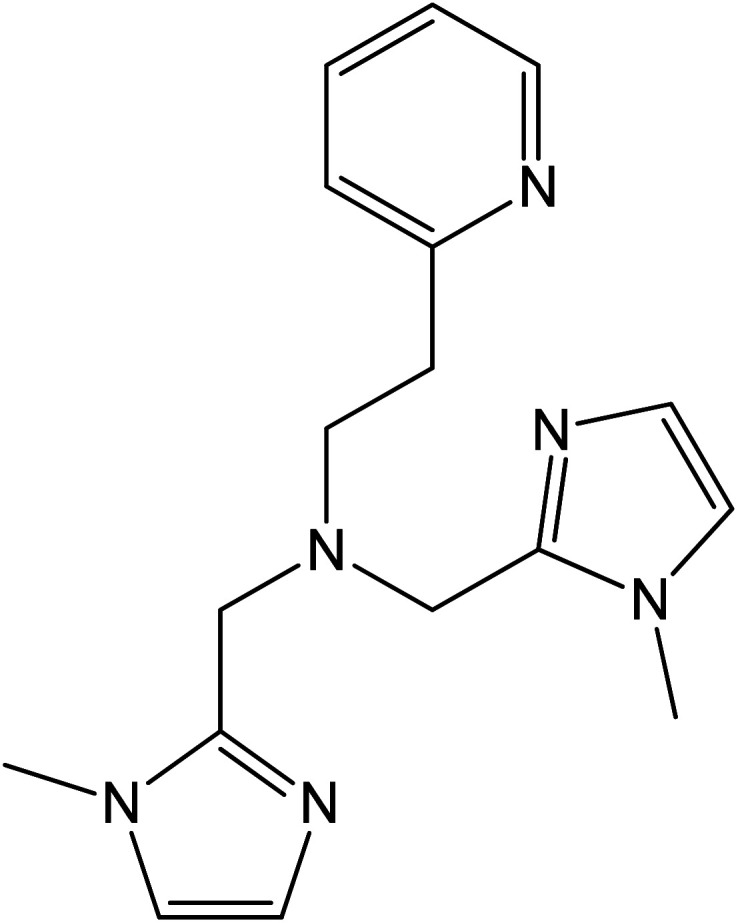
Molecular structure of the (bis(1-methylimidazol-2-yl)methyl)(2-(pyridyl-2-yl)ethyl)amine ligand (bmimapy).

These examples highlight the critical role played by the ancillary ligand in determining the redox properties of the cobalt ion inside the molecular context, and explain the relatively scarce number of these ligands available for the synthesis of VT coordination systems.^19^ Indeed, for the majority of cobalt–dioxolene complexes showing VT, these ancillary ligands can be pyridine derivatives,^39^ aromatic^40,41^ or aliphatic^40^ diazines, or tetradentate ligands, like macrocyclic derivatives of cyclam (1,4,8,11-tetraazacyclotetradecane)^42,43^ or tripodal derivatives of tpa (tris(2-pyridylmethyl)amine).^17,28,44–46^ In this work, we present the ancillary ligand bmimapy ((bis(1-methylimidazol-2-yl)methyl)(2-(pyridyl-2-yl)ethyl)amine)^47^ (see Chart 1), previously known to bind to the cobalt ion in a folded conformation^48^ and herein used to synthesize two complexes, [Co(bmimapy)(3,5-DTBCat)]PF_6_·H_2_O (1) and [Co(bmimapy)(TCCat)]PF_6_·H_2_O (2), with the objective to increase the family of nitrogen-donating, tripodal ligands for the preparation of new valence tautomeric materials.

## Results and discussion

### Synthesis

The syntheses of complexes 1 and 2 followed the previously employed route used for other 1 : 1 cobalt : dioxolene complexes featuring a tetradentated ancillary ligand:^29,44,45^ the Co^2+^ ion was coordinated by the bmimapy ligand in warm methanol under inert atmosphere, followed by the addition of the dioxolene ligand (3,5-di-*tert*-butyl-catechol (3,5-DTBCat) or tetrachlorocatechol (TCCat) for 1 and 2, respectively), deprotonated *in situ* with an excess of triethylamine. The neutral [Co(bmimapy)(diox)] complex was thus oxidized upon air exposure and precipitated with a three-fold excess of aqueous KPF_6_. Recrystallization in hot methanol yielded single crystals of the desired complexes in moderate yields.

### Structural analysis

**Fig. 1:**
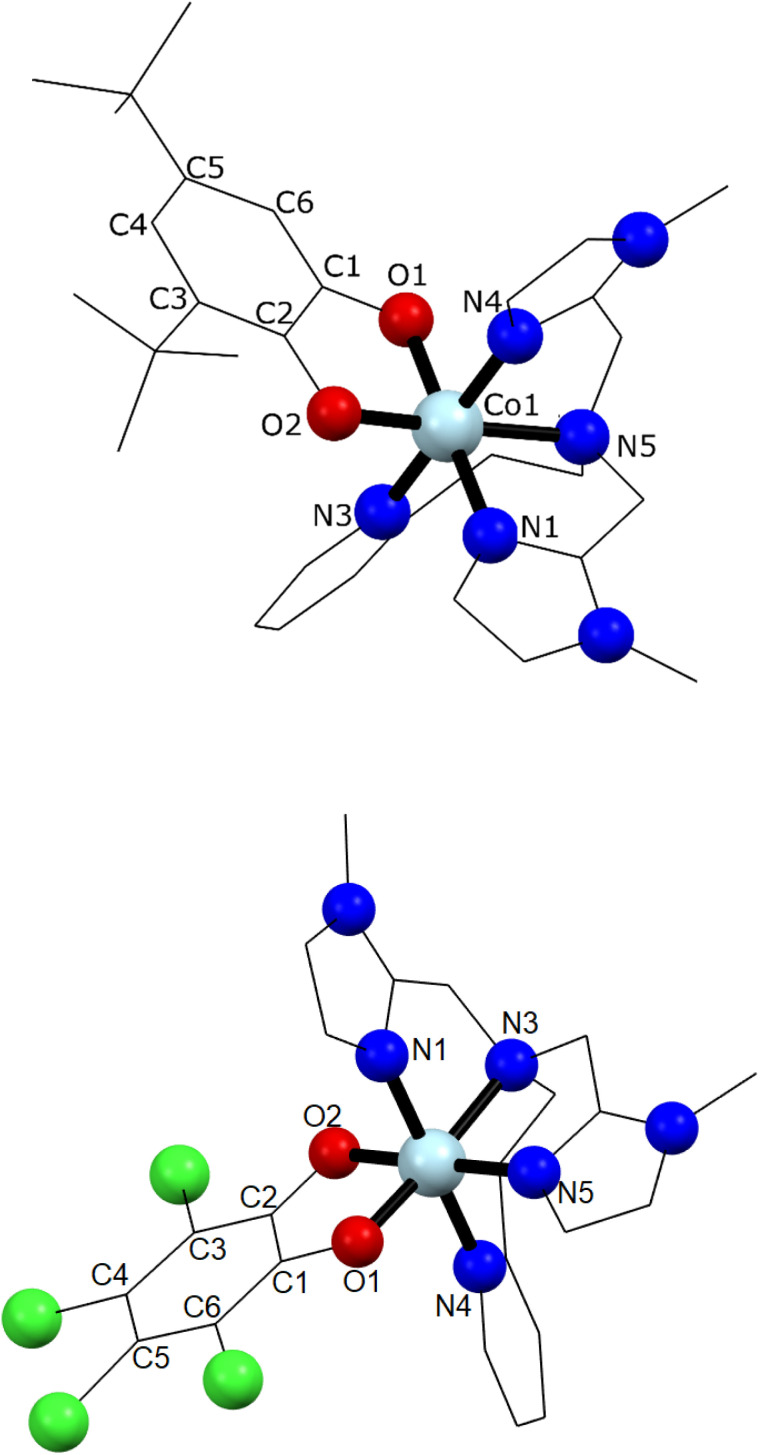
Molecular structures of complexes 1 (top) and 2 (bottom). PF_6_^−^ ion, lattice solvents, hydrogen and disordered atoms were omitted for clarity. Light blue, red, blue, green, and black colours stand for cobalt(iii) ion, oxygen, nitrogen, chlorine and carbon atoms, respectively.

Single crystal X-ray diffraction experiments were carried out at 130 K for 1 and 290 K for 2 (Fig. 1 and ESI‡). Details of data collection and structure refinement are gathered in Table S1.‡ The structure in both cases contains the cobalt ion hexacoordinated with the tetradentate ancillary ligand adopting a folded conformation around the ion, with the remaining two *cis* positions occupied by the oxygen atoms of the dioxolenes (see Fig. 1 and S1–S3‡). The coordination environment of cobalt ion is described as slightly distorted octahedral, with the continuous shape measure^49^ values of 0.396 and 0.407 for 1 and 2, respectively (Table S2‡). Co–O and Co–N bond lengths are in the 1.867–2.052 Å range, indicating a low-spin cobalt(iii) ion. Since the bond lengths of the dioxolene ligands change with the oxidation state of the ligand, C–C and C–O bond lengths can be used to calculate the metrical oxidation state (MOS) of the dioxolene.^50^ The obtained MOS values were −1.9 for both complexes, indicating that the dioxolene ligand is in the catecholate, dinegative form in both compounds. The neutrality of the systems is assured by the presence of one PF_6_^−^ anion. Selected bond lengths and bond angles are gathered in Table S3.‡

In the structure of 1, the lattice solvent molecules are disordered, and could not be successfully modelled. Therefore, the electron density of disordered solvent molecules was removed using a solvent mask procedure implemented in OLEX2.^51^ The solvent mask removed a total of 86 electrons per asymmetric unit and a volume of 360 Å^3^ which can correspond either to one water and four methanol molecules or three methanol and three water molecules. Indeed, powder X-ray diffraction of 1 shows a nearly amorphous diffractogram, presumably due to loss of lattice solvents (Fig. S4‡). The shortest intermolecular distance between metal ions is around 8.5 Å and occurs along the crystallographic direction *b*. Weak T-shaped π⋯π interactions are observed between the imidazole and pyridine moieties of one molecule and the phenyl rings of the 3,5-DTBCat ligands belonging to two surrounding molecules, yielding C–H⋯centroid distances of 2.53 and 2.87 Å, respectively. π⋯π interactions between pyridine and imidazole moieties (Fig. S2‡) also occur, with a distance between centroids of 3.763 Å and almost parallel rings, the angle between them being 8.3° and the slippage 1.681 Å. In addition, PF_6_^−^ anions interact with the complex through non-classical hydrogen bonds with intermolecular C–H⋯F distances ranging from 2.31 to 2.48 Å.^52^ In the structure of 2, one lattice water molecule and TCCat ligand are disordered and were split over two positions. The shortest intermolecular distance between metal ions is around 8.77 Å and occurs along the crystallographic direction *c*. As observed in 1, weak intermolecular interactions were observed in 2. The packing scheme also reveals C–H⋯π interactions between imidazole–imidazole rings and phenyl-imidazole rings^53,54^ (Table S4 and Fig. S3‡), with C–H⋯centroid distances of 2.78 and 2.86 Å, as well as non-classical hydrogen bonds interactions with intermolecular C–H⋯F distances ranging from 2.29 to 2.54 Å.

### Infrared spectroscopy

Infrared spectroscopy is a useful tool for determining the redox isomeric form of cobalt-based valence tautomers, due to the sensitivity of the C–O and C–C stretching energies on the oxidation state of the dioxolene ligand.^45,46,55–57^ The infrared absorption spectra of 1 and 2 (Fig. S5‡) display absorptions at 1610 and 1555 cm^−1^, typical for C=C and C–O stretching modes of dinegative catecholato as well as pyridyl ligands.^55^ The absorption peak at 1440 cm^−1^ is attributable to a mixing of C–O and C=C stretching modes, according to a previous DFT analysis of similar cobalt–dioxolene systems, and agrees with a dinegative catecholato ligands for 1 and 2.^55^ Similar conclusions can be taken considering the absorptions at 1240 (1247), 1283 (1288) and 1322 (1311) cm^−1^ for 1 (2), typical for ls-Co^III^Cat complexes.^58^ Absorptions related to the semiquinonato form of the dioxolene ligands (expected at about 1580 cm^−1^) are undetected. These data thus support a low-spin cobalt(iii)–catecholate charge distribution for 1 and 2 at room temperature in the solid state, in agreement with the structure determined by single crystal X-ray diffraction. In addition, to corroborate the cationic form of the complex, the P–F stretching and bending modes of the PF_6_^−^ anion are observed at 840 and 555 cm^−1^, respectively, for both complexes.

### Magnetometry

**Fig. 2:**
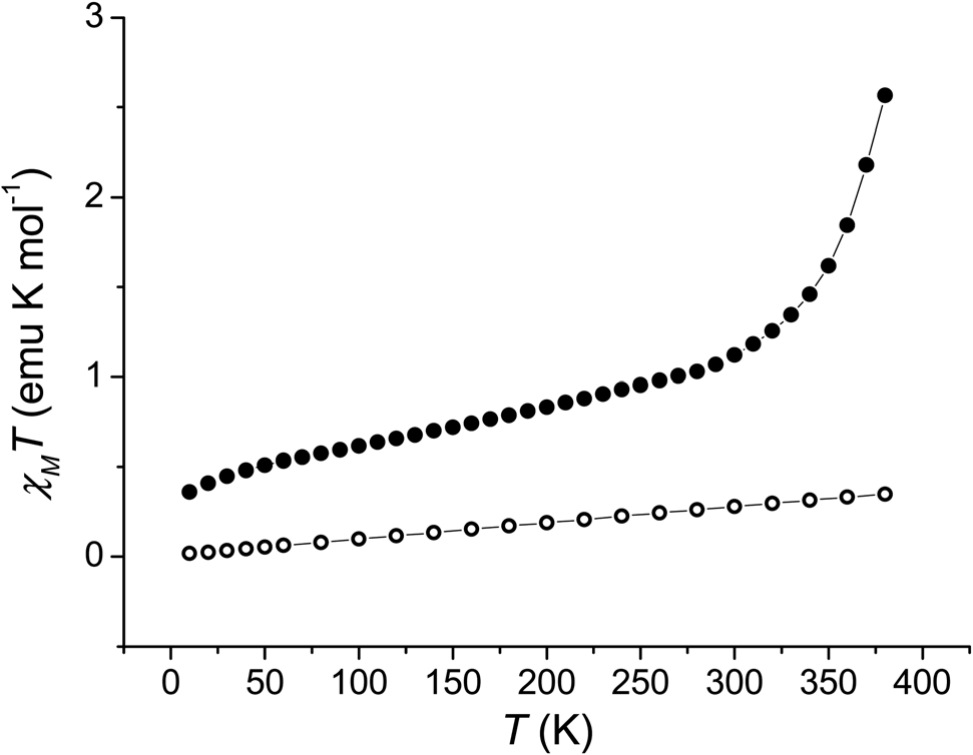
Temperature evolution of the *χ*_M_*T* product of 1 (full dots) and 2 (empty dots).

In order to quantitatively assess the charge distribution of complexes 1 and 2, the temperature dependence of their molar magnetic susceptibility (*χ*_M_) has been monitored with SQUID magnetometry in the solid state (Fig. 2).

The product of the molar magnetic susceptibility times the temperature (*χ*_M_*T*) for 1 takes the 0.36 emu K mol^−1^ value at 10 K, steadily increasing to reach 1.07 emu K mol^−1^ at 300 K. Upon heating above room temperature, a steeper increase of the *χ*_M_*T* product takes place, reaching the 2.57 emu K mol^−1^ value at 380 K. This behaviour has been attributed to the presence of a minor hs-Co^II^SQ, not interconverting, molar fraction (about 7%), and to a temperature independent paramagnetism from low-spin cobalt(iii) ion (*vide infra*), along with a gradual, incomplete, valence tautomeric transition in the 300–380 K temperature range, leading to a molar fraction of the hs-Co^II^SQ species of about 0.82. This transition profile is typical for VT transitions with low degree of cooperativity.^30^ Thermogravimetric analysis (Fig. S6‡) points out the partial loss of a solvate water molecule in this temperature window, facilitating the VT transition, as seen for other entropy driven processes.^20,59^ On the other hand, 2 displayed a linear increase of its *χ*_M_*T* product upon heating, passing from 0.02 emu K mol^−1^ at 10 K to 0.35 emu K mol^−1^ at 380 K, suggesting the presence of a temperature independent ls-Co^III^Cat charge distribution, with the diamagnetic ion showing a temperature-independent paramagnetic susceptibility of 9.04 × 10^−4^ emu mol^−1^.

### Electrochemistry

**Fig. 3:**
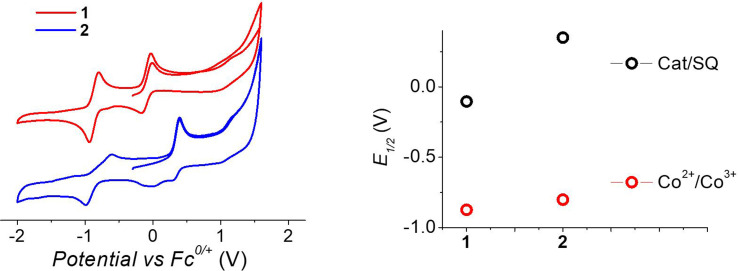
Voltammograms of acetonitrile solutions of the complexes, measured with a 100 mV s^−1^ sweeping rate, (left) and corresponding potentials (right panel).

To understand the different thermal evolution of the charge distribution in complexes 1 and 2, their electrochemical properties have been assessed with cyclic voltammetry of their acetonitrile solutions (Fig. 3).

In the case of compound 1, the first process in the anodic direction is attributed to the quasi-reversible oxidation of the catecholate ligand of the [Co(bmimapy)(3,5-DTBCat)]^+^ cation to its correspondent semiquinonate form [Co(bmimapy)(3,5-DTBSQ)]^2+^, taking place at the potential of −0.09 V (*vs.* Fc^+^/Fc redox couple, with a peak to peak separation of 140 mV, see Table S5‡). The other quasi-reversible process, going into the cathodic direction, has been attributed to the metal-centred reduction: [Co^III^(bmimapy)(3,5-DTBCat)]^+^ to [Co^II^(bmimapy)(3,5-DTBCat)], and features a potential of −0.86 V (*vs.* Fc^+^/Fc redox couple), with a peak to peak separation of 140 mV. In the case of compound 2, a similar potential is observed for the Co^2+/3+^ redox couple (−0.80 V), supporting the interpretation of a metal-centred electrochemical process. The oxidation of the tetrachlorocatechol moiety, on the other hand, occurs at significantly higher potential (+0.35 V), as expected considering the electron-withdrawing nature of the chlorine substituents on the catechol ring.

The metal-centred process can be used to understand the thermodynamic origin of the entropy-driven VT process in 1. It is in fact known that the reduction potential of the 3,5-di-*tert*-butyl-catecholate ligand in [M^II^(L)(3,5-DTBSQ)]^+^/[M^II^(L)(3,5-DTBCat)] complexes is, to a first approximation, dependent on the oxidation state of the metal ion, but does not depend on its chemical identity, its spin state, as well as on the nature of the ancillary ligand L.^28^ We can thus assume this reduction potential to be similar to the one observed for the [Co^2+^(Me_3_tpa)(3,5-DTBSQ)]^+^ cation, found to be −0.78 V (*vs.* Fc^+^/Fc).^28^ We can thus estimate the difference between the free energy of the [Co^III^(bmimapy)(3,5-DTBCat)]^+^ and [Co^II^(bmimapy)(3,5-DTBSQ)]^+^ redox isomers 
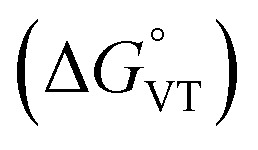
 of 1 as the difference between the potential of the Co^3+/2+^ and the SQ/Cat redox couples:^28^
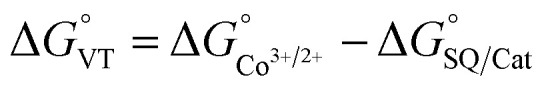


The obtained value of 8 kJ mol^−1^ for 
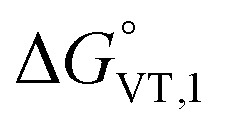
 is in the range observed for a system undergoing valence tautomerism.^28^ Taking the oxidation potential of the [Ni(bdi)(TCCat)] complex^60^ (+0.2 V, being bdi the *N*,*N*′-bis(2,4,6-trimethylphenyl)-2,3-butanediimine) as reference for the TCCat/TCSQ redox couple, we find a 
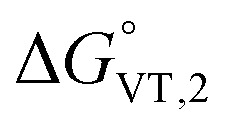
 of +96 kJ mol^−1^ for complex 2, thus justifying its observed temperature independent low-spin cobalt(iii)–catecholate charge distribution. In a similar approach, Boskovic and co-workers related the spacing between the metal- and dioxolene-centred electrochemical processes in the same molecule (*Δ*_ox–red_) to the occurrence of VT, identifying a threshold of 740 mV, below which the VT occurs in the 10–300 K range.^61^ Our data fit fairly well into this treatment, with 1 displaying a *Δ*_ox–red_ of 770 mV, corresponding to a VT entropy-driven transition above room temperature.

### DFT analysis

**Fig. 4:**
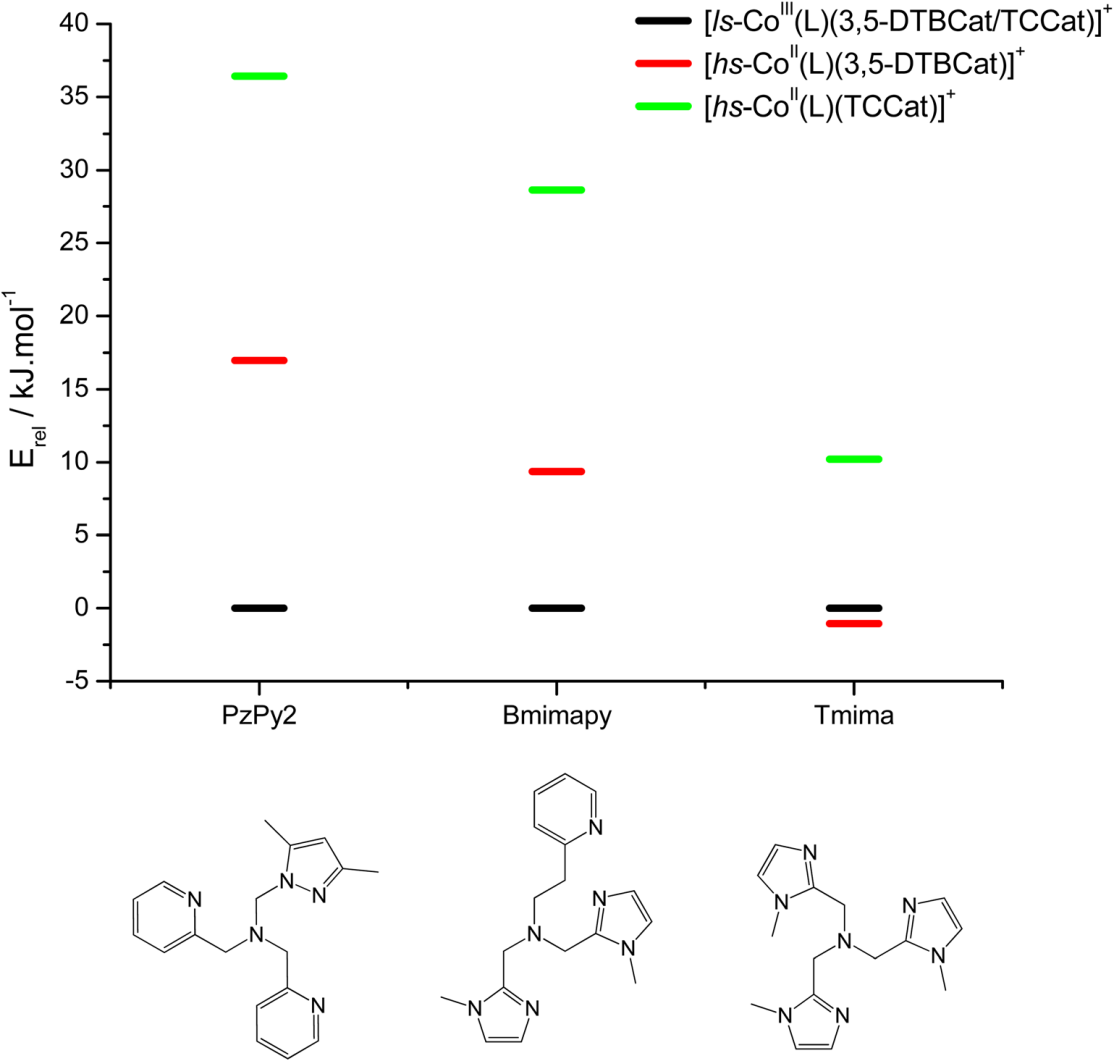
DFT calculated relative energies of the different charge distribution electrometric forms of [Co(L)(diox)]PF_6_ complexes.

DFT has been previously used to interpret and predict the potential VT behaviour of cobalt complexes.^37,61,62^ We have employed the TPSSh functional with the def2-TZVP basis set to calculate the relative energy differences (*E*_rel_) between ls-Co^III^Cat and hs-Co^II^SQ redox isomers for several complexes with the [Co(L)(diox)]PF_6_ formula, with the aim to clarify the role of the bmimapy ligand in tuning the Co^2+/3+^ redox potential. Indeed, the TPSSh functional has been successfully employed in the recent past to calculate such energy differences in VT cobalt complexes.^38,62,63^ Here, the diox ligand alternates between the 3,5-di-*tert*-butyl-catecholate or the tetrachlorocatecholate ligands, while three ancillary ligands L were chosen to represent the effect of the imidazole binding groups of the bmimapy ligand: PzPy_2_ (ref. 34) ((3,5-dimethyl-1*H*-pyrazol-1-yl)-*N*,*N*-bis(pyridin-2-ylmethyl)methanamine, known to provide [Co(L)(diox)]^+^ cations showing the ls-Co^III^Cat charge distribution), bmimapy and tmima (tris[(1-methyl-2-imidazolyl)methyl]amine),^64,65^ representing the limit of a tripodal ligand featuring a central nitrogen atom bound to three 1-methyl-imidazole pendant arms. The results of the DFT analysis are reported in Fig. 4, S7 and Table S6.‡ A semi-quantitative evaluation of the solidity of our approach can be obtained observing the results for the [Co(Me_3_tpa)(3,5-DTBSQ)]^+^ (ref. 28) and [Co(Me_3_tpa)(TCSQ)]^+^ (ref. 29) complexes (Fig. S8‡), which parallel the experimentally observed charge distribution in literature. As such, we expect an uncertainty of about ±4 kJ mol^−1^ on the *E*_rel_ value. It must be stressed that *E*_rel_ does not consider the different vibrational structure of the two electromers and cannot, as such, be considered an estimation of 
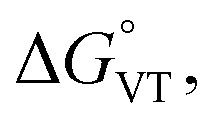
 but simply an estimation of the enthalpic difference between the two electromeric forms.^61^

The comparison between the two families of complexes containing the 3,5-DTBCat and TCCat ligands shows a significantly higher *E*_rel_ for the latter, as expected. Moreover, the calculated energy difference between the 3,5-DTBCat and TCCat derivatives is almost independent of the ancillary ligand L, as previously pointed out, supporting the assumptions we made in the discussion of the electrochemical properties of 1 and 2. The effect of the ancillary ligand, on the other hand, can be understood comparing the trend found for each of these two families of complexes. Considering the 3,5-DTBCat family, the highest value of *E*_rel_ is found for the complex with the PzPy_2_ ligand. This result complies with the ls-Co^III^Cat charge distribution experimentally observed for the [Co(PzPy_2_)(3,5-DTBCat)]^+^ complex,^34^ thus acting as an additional proof of the solidity of our computational results. Moving to the cases of the cobalt complexes of the 3,5-DTBCat dioxolene including the bmimapy and tmima ligands, the calculated *E*_rel_ values decrease. For the bmimapy derivative (compound 1), the DFT evaluation of *E*_rel_ (9.4 kJ mol^−1^) is in very good agreement with the 
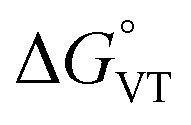
 value extrapolated from the electrochemical analysis, justifying the incipient, high temperature valence tautomeric process observed experimentally. For the tmima derivative, moreover, such a stabilisation leads to a hs-Co^II^SQ electronic ground state suggested for the [Co(tmima)(3,5-DTBCat)]^+^ derivative (yet unreported in literature). This comparison highlights that the methyl-imidazole arm of the ligands favours the reduced form of the metal building-block, thus lowering the *E*_rel_. This finding cannot be attributed to steric effects, since the imidazole arm generates a five-membered coordination ring, similarly to the methylene-pyridine and methylene-pyrazole arms of the PzPy_2_ ligand. Moreover, no sterically demanding groups are present in the *ortho* position of the heterocyclic groups, differently from the case of the pyrazole arm of the PzPy_2_ ligand, bearing a methyl group in *ortho* with respect to the donor nitrogen atom. Thus, we attribute this behaviour to the higher π-donation ability of the imidazole ligands, when compared with the pyridine rings. Indeed, pyridines are known to possess low energy π* orbitals able to accept back-donation from the cobalt ion, thus stabilising the higher Co^3+^ oxidation state.^66^ In the case of the family of complexes featuring the tetrachlorocatecholate ligand, a similar trend is observed, confirming the relative electrochemical independence of one redox-active building-block (Co(L)) from the other (the dioxolene anion) and corroborating our DFT analysis.

## Conclusions

This works introduces the tripodal ancillary bmimapy ligand to the community of switchable molecular materials for the preparation of valence tautomeric complexes. Its reactivity is similar to other ancillary ligands used in the literature to prepare heteroleptic cobalt complexes, bonding to the cobalt(ii) ion in a folded conformation, thus leaving two *cis* positions for the coordination of the catecholate chelating ligands. Bmimapy was used to prepare two complexes: [Co(bmimapy)(3,5-DTBCat)]PF_6_·H_2_O (1) and [Co(bmimapy)(TCCat)]PF_6_·H_2_O (2), where 3,5-DTBCat and TCCat are the 3,5-di-*tert*-butyl-catecholate and tetrachlorocatecholate anions, respectively. Single crystal X-ray diffraction analysis reported a distorted octahedral coordination environment for the cobalt ions in both complexes, and the bond lengths of the first coordination sphere, including also the MOS analysis of the bond lengths of the catecholate moieties, indicated a ls-Co^III^Cat charge distribution for both complexes. In the 300–380 K range, however, magnetic susceptibility highlighted an incipient, valence tautomeric process for 1, while 2 retained a ls-Co^III^Cat charge distribution. Cyclic voltammetry allowed to understand this behaviour, calculating the free energy difference associated to the VT process for both complexes, which resulted in a range accessible with thermal stimula above room temperature for 1 (8 kJ mol^−1^), while it did not for 2 (96 kJ mol^−1^). These experimental observations were finally justified by a DFT analysis, highlighting the ability of the methyl-imidazole pendant arm of the bmimapy ligand to stabilise the high-spin Co(ii) redox form and favouring the onset of the VT phenomenon in bmimapy containing cobalt complexes. The chemical control of the reduction potential of different metal ions provided by the bmimapy ligand is expected to be a powerful tool to increase the library of valence tautomeric complexes.

## Conflicts of interest

There are no conflicts of interest to declare.

## Supplementary Material

RA-013-D3RA03235C-s001

RA-013-D3RA03235C-s002
