# Supplementary material for: Valence tautomerism in a cobalt–dioxolene complex containing an imidazolic ancillary ligand
Source: RSC Adv. 2023 Jul 4;13(29):20050–7. doi: 10.1039/d3ra03235c (PMC10318486; doi:10.1039/d3ra03235c)
Supplement: RA-013-D3RA03235C-s001 [file RA-013-D3RA03235C-s001.pdf]

Electronic Supplementary Material (ESI) for RSC Advances.
This journal is © The Royal Society of Chemistry 2023

ELECTRONIC SUPPLEMENTARY INFORMATION

Valence Tautomerism in a Cobalt-dioxolene complex containing an imidazolic ancillary ligand

Anderson Moledo Vicente Guedes, Leandro Sodré de Abreu, Igor Antunes Vogel Maldonado, William Silva Fernandes, Thiago Messias Cardozo, Rafael A. Allão Cassaro, Marciela Scarpellini, Giordano Poneti[#*]

Instituto de Química, Universidade Federal do Rio de Janeiro, Rio de Janeiro, RJ, 21941-909, Brazil

[#]*current address: Dipartimento di Scienze Ecologiche e Biologiche, Università degli Studi della Tuscia, Largo dell'Università, 01100, Viterbo, Italy*

- Corresponding author giordano.poneti@unitus.it

Experimental Section.

Materials and instrumentation.

FTIR spectra on solid samples were recorded with an ATR - Shimadzu-IRaffinity1 spectrometer. Elemental analysis (C, H, N) was performed with a Perkin Elmer 2400 series II analyser. Thermogravimetry analysis was recorded on a TGA-50 Shimadzu. The sample was heated from 25-600 °C at 10° C min^{-1} rate under argon flux of 50 mL min^{-1}.

Syntheses.

Unless indicated otherwise, all manipulations were performed under aerobic conditions using materials as received. $CoCl_2·6H_2O$, 3,5-ditertbutyl-catechol (3,5-DTBCat), tetrachlorocatechol (TCCat) and triethylamine were purchased from Aldrich and used as received. The ancillary ligand [(bis(1-methylimidazol-2-yl)methyl)(2-(pyridyl-2-yl)ethyl)amine] (bmimapy) was synthesized according to previously reported procedure.[1]

[Co(bmimapy)(3,5-DTBCat)]$PF_6·H_2O$ **(1)**. A methanol solution of bmimapy (0.07 g, 0.22 mmol) was added to a stirring methanol solution of $CoCl_2·6H_2O$ (0.048 g, 0.20 mmol) under N_2 atmosphere. To this mixture a methanol solution of 3,5-DTBCat (0.04 g, 0.20 mmol) and triethylamine (705 µl, 5.07 mmol) was added under N_2 atmosphere. A colour changing from dark purple to dark blue was observed. After 30 minutes of refluxing, the methanol solution was exposed to the atmosphere, filtered, and an aqueous solution of KPF_6 (0.11 g, 0.61 mmol) was added to the filtrate, which was subsequently heated for further 15 minutes. Upon cooling, a green precipitate formed which was collected by filtration and recrystallised from warm methanol, yielding 67 % of small crystals. The crystals are unstable out of mother solution under ambient conditions presumably due to quick desolvation of lattice solvents. Such finding is confirmed by elemental analysis and thermogravimetry (TGA), showing a loss of around 1% of mass starting from ambient temperature to 77 °C (vs an expected 2.4%, on the basis of the crystallographic analysis, see Figure S6), indicating weak interacting lattice solvents. Anal. Calcd for $C_{31}H_{44}CoF_6N_6O_2P$: C, 49.47; H, 5.89; N, 11.16. Found: C, 49.66; H, 5.78; N, 10.73 %. Selected IR data (cm^{-1}): 3450(m), 3129(w), 2955(m), 2862(w), 1612(w), 1566(w), 1524(m), 1441(s), 1415(m), 1321(w), 1283(m), 1242(m), 1178(w), 1110(w), 1084(s), 1028(s), 979(m), 840(vs), 752(m), 662(w), 621(s), 558(s), 467(w).

[Co(bmimapy)(TCCat)]$PF_6·H_2O$ **(2)**. The same procedure was followed, replacing 3,5-ditertbutyl-catechol with tetrachlorocatechol. Yield: 72 %. Slowly desolvation occurs for **2** at ambient conditions. In fact, the elemental analysis result is in good agreement with the one expected for a desolvated sample. Anal. Calcd for $C_{23}H_{22}Cl_4CoF_6N_6O_2P$: C, 36.24; H, 3.17; N, 11.03. Found: C, 36.40; H, 3.17; N, 11.41

%. Selected IR data (cm^{-1}): 3450(m), 3145(w), 1612(w), 1524(m), 1442(vs), 1372(m), 1309(w), 1290(m), 1253(s), 1218(w), 1177(m), 1117(w), 1079(w), 1023(w), 975(m), 919(s), 840(vs), 813(m), 763(m), 736(s), 686(s), 662(s), 595(s), 558(vs), 531(w), 444(w).

Cyclic Voltammetry.

The redox behavior of the studied complexes was investigated by cyclic voltammetry using an Epsilon potentiostat-galvanostat from Bioanalytical Systems (BAS). The experiments were carried out in spectroscopic grade acetonitrile (UV/HPLC) using tetrabutylammonium hexafluorophosphate ($TBAPF_6$) (0.1 mol L^{-1}) as supporting electrolyte, under inert atmosphere (argon). An electrolytic cell composed by three electrodes was used: working electrode – vitreous carbon; auxiliary electrode – platinum wire; reference pseudo-electrode – Ag/AgCl in acetonitrile containing $TBAPF_6$ 0.1 mol L^{-1}. To monitor the reference *pseudo*-electrode, the redox pair ferrocenium/ferrocene (Fc^+/Fc) was used as reference standard. Potential values are expressed in relation to the Fc^+/Fc pair. The working potential range used was -1800 to +1800 mV and the sweep speed 100 mV s^{-1}.

Magnetometry.

Samples employed for magnetic measurements consisted of pressed microcrystalline powders of **1** and **2**, wrapped in Teflon(TM) tape. The magnetic characterization was performed on Quantum Design MPMS (Magnetic Properties Measurement System) equipment provided with a 5 T magnet. The magnetization (M) dependence with the absolute temperature was investigated between 380 and 60 K using a magnetic field (B) of 10 kOe, and between 60 and 10 K with a field of 1 kOe to prevent magnetic saturation. After subtraction of the diamagnetic contribution of the sample holder and the sample, evaluated with Pascal's constants,[12] magnetic susceptibility per mole (χ_M) was evaluated as $\chi_M = M_M/B$.

Thermogravimetric Analysis.

The thermogravimetric curve was measured with a Shimadzu DTG-60 Analyzer, applying a 10 K min^{-1} scan rate, under a 50 mL min^{-1} flow rate of argon.

X-ray diffraction studies

Single crystal X-ray diffraction data for both complexes was collected on a Bruker D8 Venture diffractometer using Mo K_α radiation (λ = 0.71073 Å). Data collection, data reduction and cell

refinement were performed by using the Bruker Instrument Service v4.2.2 and SAINT V8.34A softwares.[2] The structure was solved by direct methods using the SHELXS program, and refinement was performed using SHELXL based on F^2 through full-matrix least squares routine[3] within winGX package.[4] Empirical multi-scan absorption corrections using equivalent reflections were performed with SADABS program.[5] All non-hydrogen atoms were refined with anisotropic displacement parameters. Hydrogen atoms were set in calculated positions and refined using the riding model.[6] The structures were drawn using the Mercury software.[7] Compound **1** show instability losing its diffracting properties if the crystal is kept out of mother liquor at ambient conditions. This occurs presumably due to partial or total loss of the lattice solvents. For this reason, low completeness data was obtained. Lattice water molecule, tetrachlorocatechol ligand and PF_6^- anion are disordered in **2.** The first two are disordered over two with positions. Atoms occupancies were freely refined. DFIX restraint was applied to P-F bond length in PF_6^- anion. SADI command was applied in order to have similar bond lengths for C-C and C-O in the tetrachlorocatechol ligand. Anisotropic displacement parameters of some atoms of catechol were restrained to be the same and phenyl ring was restrained to be flat.

DFT Calculations

The computational studies of the complexes were carried out with DFT level theory, using TPSSh functional, def2-TZVP basis function and D3 dispersion interaction energy correction in ORCA 5.0.3 quantum package.[9-11] Every geometry of each structure was optimized to an energy minimum in PES, both for the singlet *ls*-Co^{3+}Cat state and for the quintet *hs*-Co^{2+}SQ state of each complex.

Table S1: Summary of data collection and crystal structure refinement for **1-2**.

	**1**	**2**
Formula	$C_{31}H_{42}F_6N_6O_2P_1Co_1$+[Solvent]	$C_{23}H_{24}N_6O_3Cl_4F_6P_1Co_1$
Formula weight (g mol^{-1})	734.60	778.18
Temperature (K)	132(2)	291(2)
Wavelength (Å)	0.71073	0.71073
Crystal system	Monoclinic	Monoclinic
Space group	$P2_1/c$	$P2_1/c$
a (Å)	12.945(4)	8.8538(5)
b (Å)	15.221(5)	19.1183(10)
c (Å)	22.683(10)	17.5365(8)
a (°)	90	90
β (°)	100.93(2)	93.149(2)
γ (°)	90	90
V ($Å^3$)	4388(3)	2963.9(3)
Z	4	4
ρ_{calc} (Mg m^{-3})	1.339	1.739
μ (mm^{-1})	0.505	1.071
F(000)	1528	1560
θ range (Deg.)	2.97- 25.02	2.130 - 25.680
Index ranges	$-14<=h<=16$<br>$-18<=k<=16$<br>$-14<=l<=28$	$-10<=h<=10$<br>$-23<=k<=23$<br>$-21<=l<=21$
Data collected/ Independent reflections	9405/6125	99627/5626
R_{int}	0.0803	0.0624
Refinement method	Full-matrix least-squares on F^2	
Data / restraints / parameters	6125/ 16 / 418	5626 / 38 / 574
GOF on F^2	1.024	1.073
$R_1;wR_2$ [$I>2\sigma(I)$]	0.1015, 0.2878	0.0384, 0.0878
$R_1;wR_2$ (all)	0.1598, 0.3425	0.0510, 0.0964
$\Delta\rho_{max}; \Delta\rho_{min}$ (e·$Å^{-3}$)	1.18, -1.20	0.592, -0.443

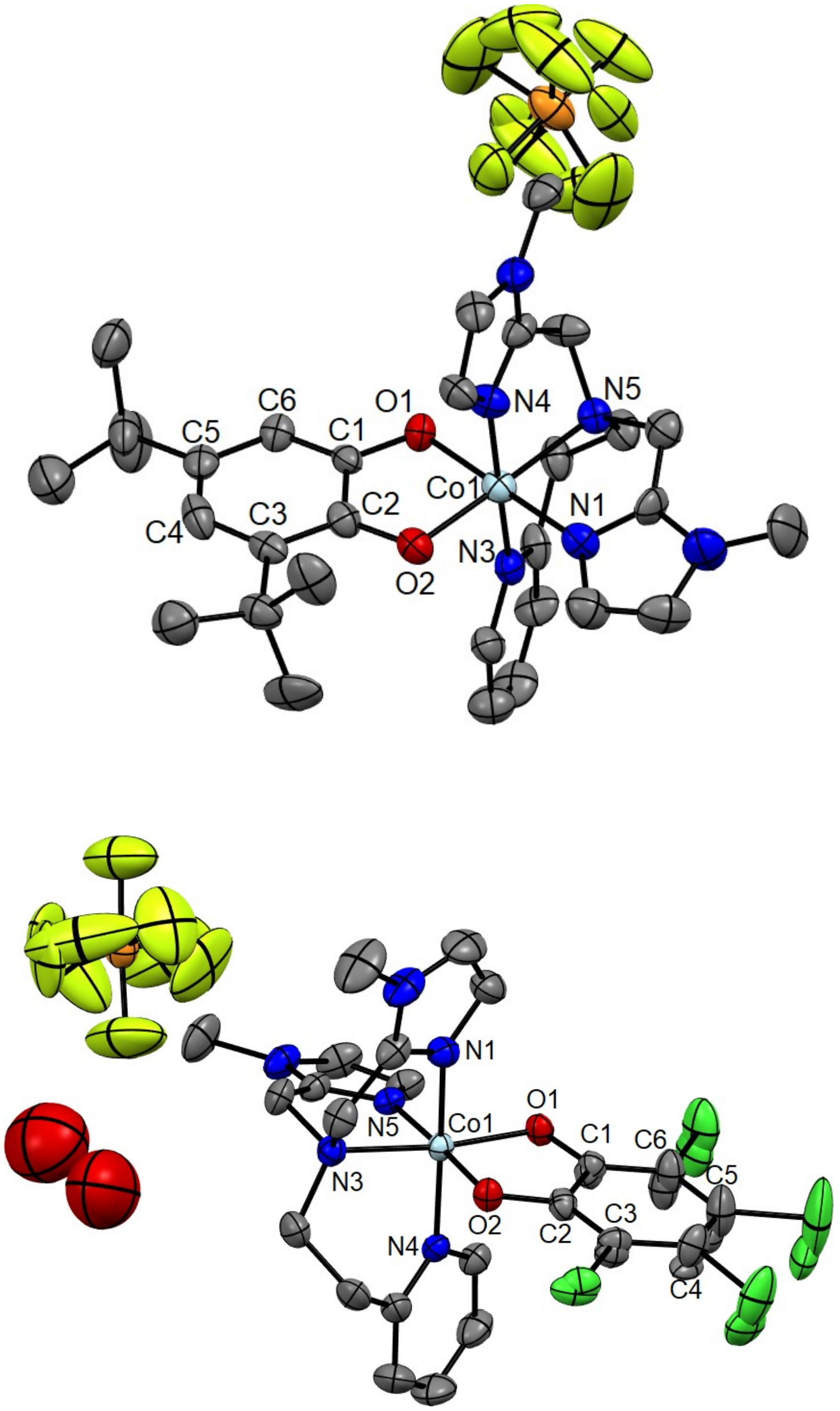

Figure S1: Asymmetric unit of **1** (top) and **2** (bottom). The displacement ellipsoids are drawn at 50 % of probability. Hydrogen atoms were omitted for clarity. Light blue, red, blue, green, neon green, orange and dark grey colour stands for cobalt(III) ion, oxygen, nitrogen, chlorine, fluorine, phosphorus and carbon atoms, respectively.

Table S2: Continuous Shape Measures Calculations of the coordination spheres of **1** and **2**.

Compound	**OC-6**	TPR-6	JPPY-6	HP-6	PPY-6
**1**	**0.396**	14.499	29.798	30.293	26.504
**2**	**0.407**	14.553	29.459	29.707	26.161

OC-6 Octahedron, TPR-6 Trigonal prism, JPPY-6 Johnson pentagonal pyramid J2, HP-6 Hexagon, PPY-6 Pentagonal pyramid

Table S3: Selected bond lengths and bond angles for **1** and **2**.

**1**		**2**	
**Bond Length (Å)**			
N1-Co1	1.930(7)	N1-Co1	1.919(2)
N3-Co1	1.958(6)	N3-Co1	2.040(2)
N4-Co1	1.912(6)	N4-Co1	1.948(2)
N5-Co1	2.052(7)	N5-Co1	1.919(2)
O1-Co1	1.889(5)	O1-Co1	1.8678(18)
O2-Co1	1.867(6)	O2-Co1	1.8928(18)
O1-C1	1.336(9)	O1-C1	1.274(14)
O2-C2	1.336(10)	O2-C2	1.320(17)
C1-C2	1.430(11)	C1-C2	1.422(7)
C3-C2	1.383(12)	C3-C2	1.397(7)
C3-C4	1.432(12)	C3-C4	1.408(9)
C5-C4	1.368(13)	C5-C4	1.384(9)
C5-C6	1.384(12)	C5-C6	1.394(9)
C1-C6	1.362(11)	C1-C6	1.386(8)
**Bond Angle (°)**			
O1-Co1-N1	179.1(3)	O1-Co1-N1	92.26(9)
O1-Co1-N4	87.9(3)	O1-Co1-N4	90.75(9)
O1-Co1-N5	95.3(2)	O1-Co1-N5	93.74(8)
O1-Co1-O2	88.3(3)	O1-Co1-O2	88.16(8)
O2-Co1-N1	92.5(3)	O2-Co1-N1	87.07(9)
O2-Co1-N3	91.1(3)	O2-Co1-N3	94.47(8)
O2-Co1-N4	92.3(3)	O2-Co1-N4	88.70(9)
N1-Co1-N3	91.5(3)	N1-Co1-N3	81.12(10)
N4-Co1-N3	174.6(3)	N4-Co1-N3	96.09(9)
N5-Co1-N3	95.5(3)	N5-Co1-N3	83.78(9)
N5-Co1-N1	84.0(3)	N5-Co1-N1	94.03(9)
N5-Co1-N4	81.3(3)	N5-Co1-N4	90.09(9)
N1-Co1-N4	92.5(3)	N1-Co1-N4	174.73(10)
O1-Co1-N3	88.1(2)	O1-Co1-N3	172.72(9)
O2-Co1-N5	172.6(2)	O2-Co1-N5	177.76(9)

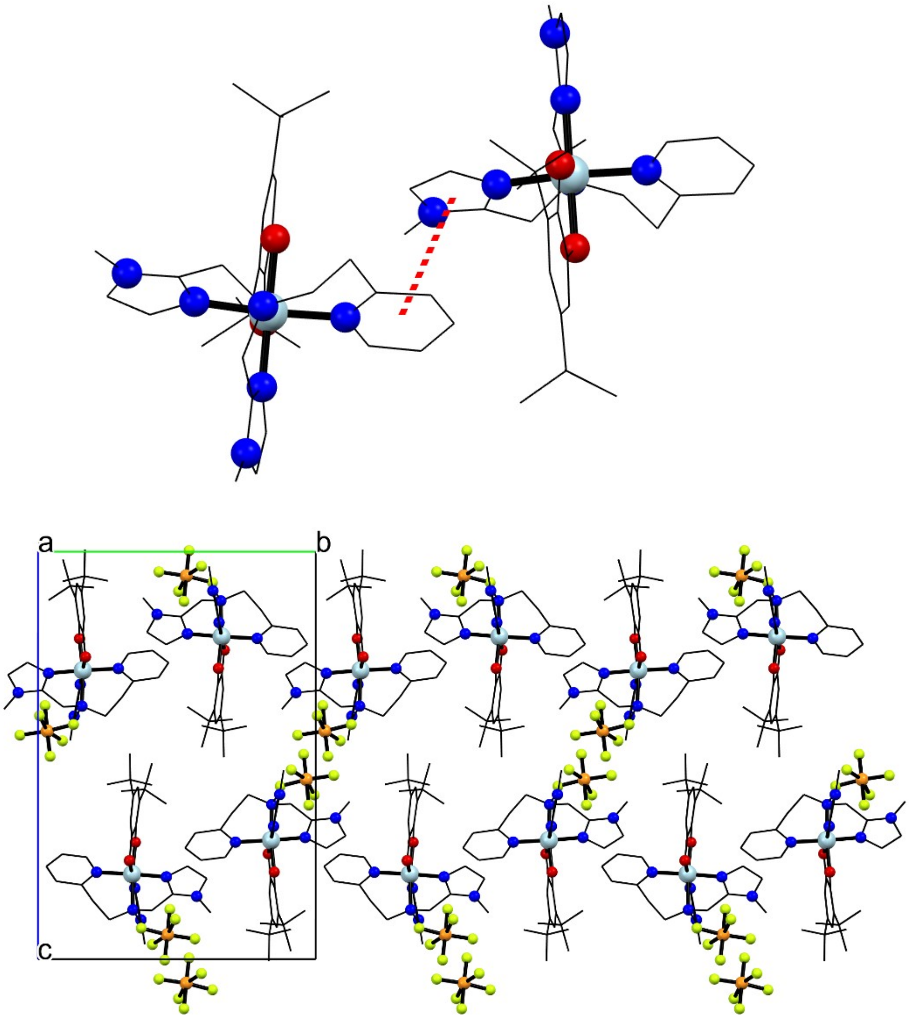

Figure S2: View of compound **1** emphasizing π···π stacking between adjacent units (top). Crystal packing view along *a* direction (bottom). Hydrogen atoms were partially omitted for clarity.

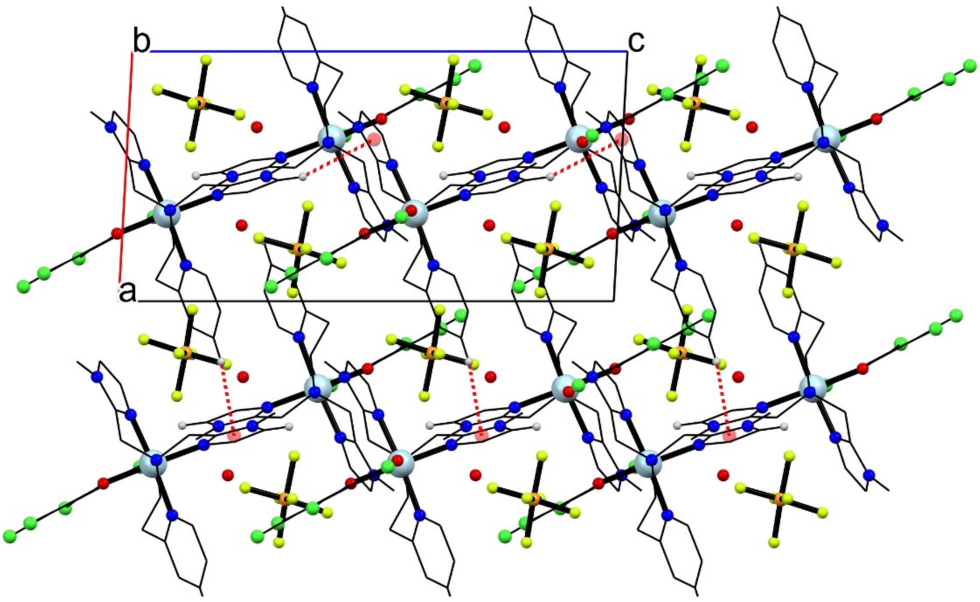

Figure S3: Crystal packing of **2** viewed along *b* direction. Hydrogen atoms were partially omitted for clarity. The C-H···π interactions are represented by dashed red lines.

Table S4: C-H···π interaction parameters for **2**.

	H···Cg (Å)	H-perp (Å)[a]	γ(°)[b]	C-H...Cg (°)	C...Cg (Å)	Symmetry Operation[c]
C22-H58···Cg1[c]	2.86(4)	2.73	16.92	156(3)	3.708(3)	x, 1/2-y, 1/2+z
C17-H63···Cg2[c]	2.78(4)	2.75	7.86	157(3)	3.617(4)	1+x, y, z

[a]Perpendicular distance of H to ring plane, [b]Angle between Cg-H vector and ring normal, Cg is the centroid of the ring.

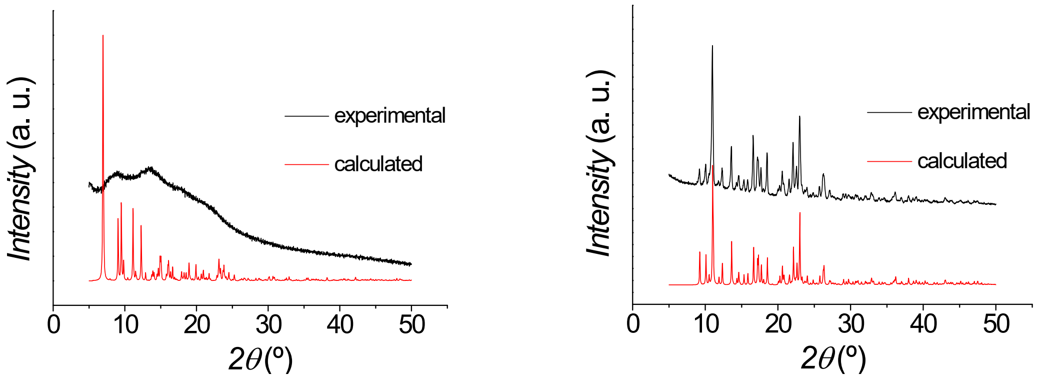

Figure S4: Powder X-ray diffraction patterns of **1** (*left*) and **2** (*right*).

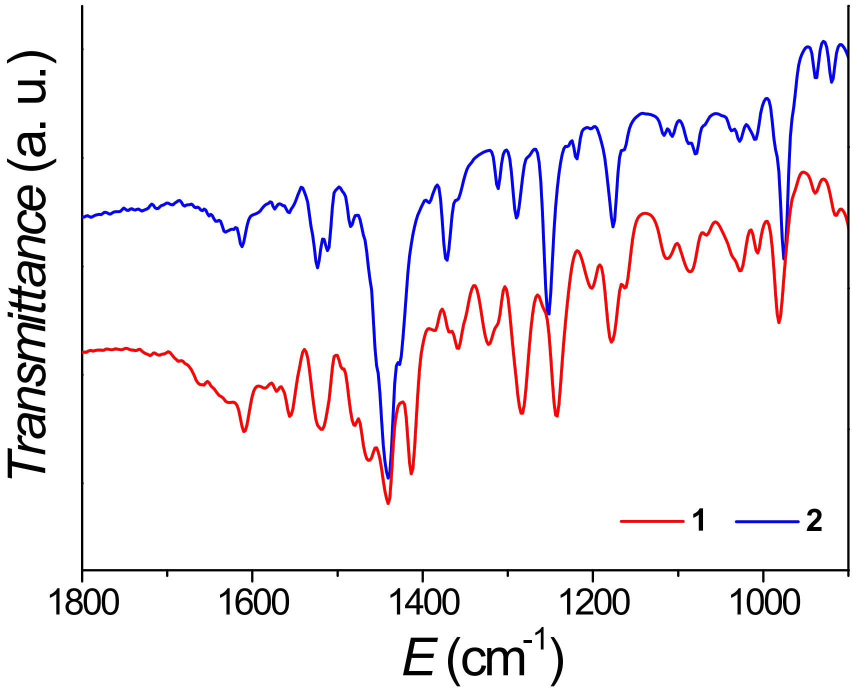

Figure S5: Infrared spectra of **1** and **2**, measured at room temperature on pressed KBr pellets.

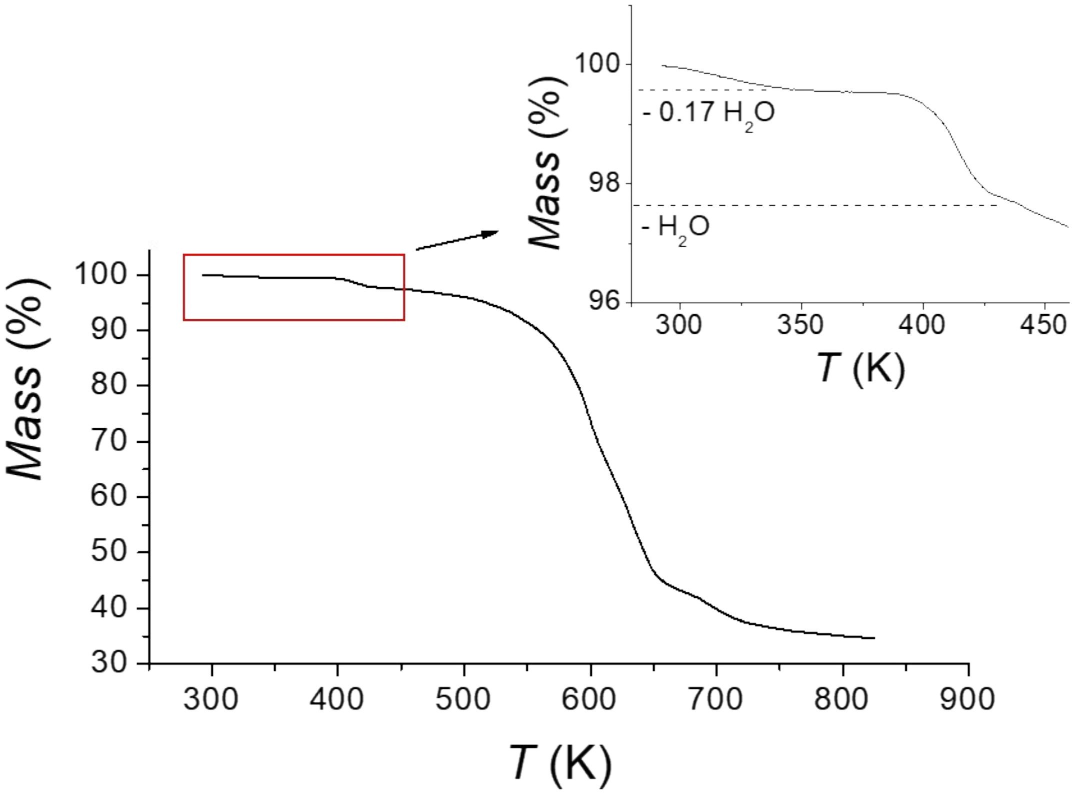

Figure S6: Thermogravimetric analysis showing the loss of the lattice solvent (a molecule of water) in **1** in the 300 – 450 K range.

Table S5: Electrochemical data of complexes **1** and **2**, extracted from cyclic voltammetry of their acetonitrile solutions (1.0 mM analyte with 0.1 M Bu_4NPF_6, scan rate 100 mVs^{-1}). Potentials are reported in volts *vs* the Fc^+/Fc redox couple. E_{pc}, E_{ac} and $E_{1/2}$ report, respectively, the cathodic, anodic and half-wave potentials. *Δox-red* is the difference of the half-wave potentials of the metal- and ligand-centred processes.[8] Peak-to-peak separations (*ΔE*) and *Δox-red* are reported in mV.

	Metal-based process			*Ligand-based process*			*Δox-red*
	[**Co^II^**(bmimapy)(Cat)]$^+$/ [**Co^III^**(bmimapy)(Cat)]			[Co^{III}(bmimapy)(**Cat**)]$^+$/ [Co^{III}(bmimapy)(**SQ**)]			
	E_{pc}	E_{ac}	$E_{1/2}$ (*ΔE*)	E_{pc}	E_{ac}	$E_{1/2}$ (*ΔE*)	
**1**	-0.93	-0.79	-0.86(140)	-0.16	-0.02	-0.09 (140)	770
**2**	-0.99	-0.61	-0.80(380)	0.31	0.39	0.35 (80)	1150

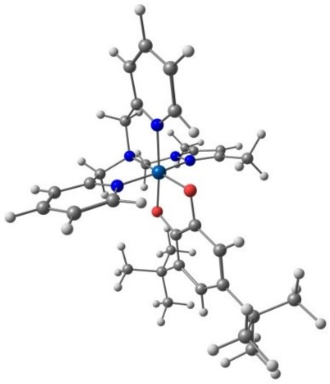

$[Co(PzPy_2)DTBCat]^+$

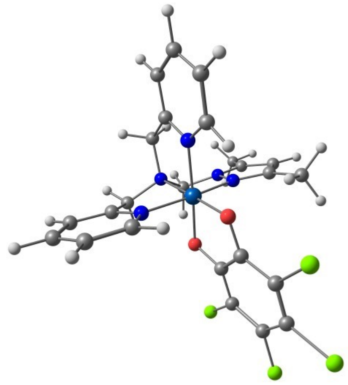

$[Co(PzPy_2)TCCat]^+$

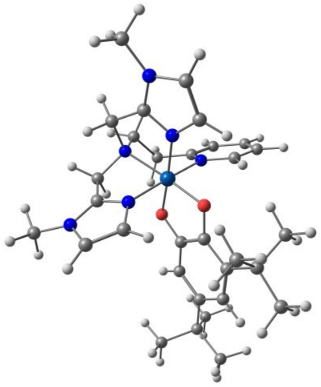

$[Co(BMIMAPY)DTBCat]^+$

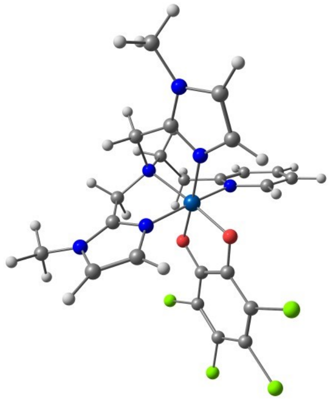

$[Co(BMIMAPY)TCCat]^+$

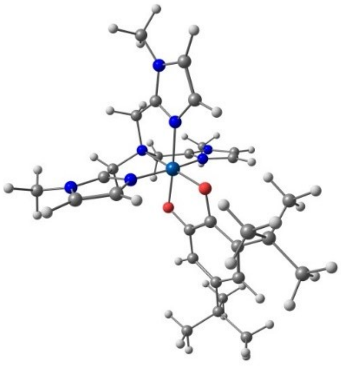

$[Co(TMIMA)DTBCat]^+$

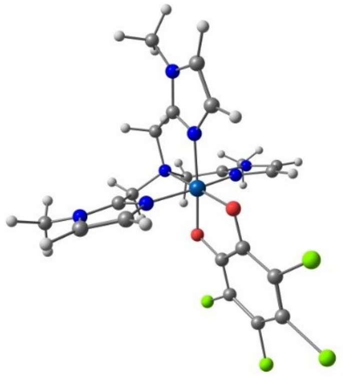

$[Co(TMIMA)TCCat]^+$

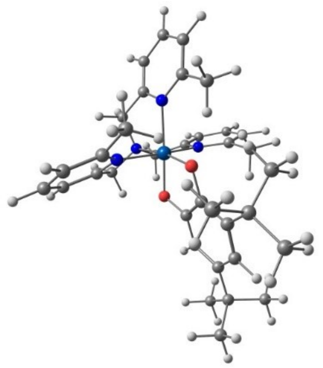

[Co(Me_3TPA)DTBCat]$^+$

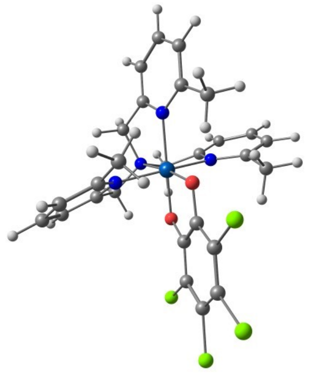

[Co(Me_3TPA)TCCat]$^+$

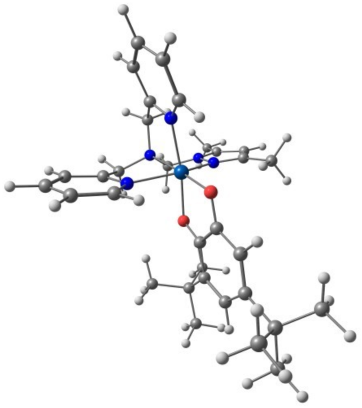

[Co($PzPy_2$)DTBSQ]$^+$

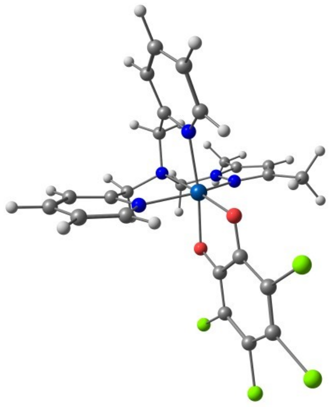

[Co($PzPy_2$)TCSQ]$^+$

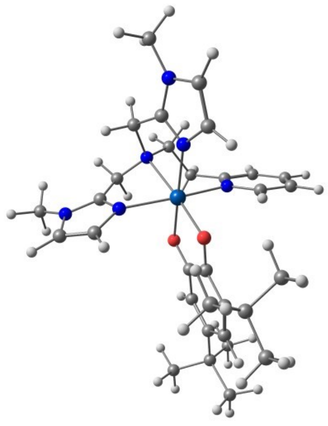

[Co(BMIMAPY)DTBSQ]$^+$

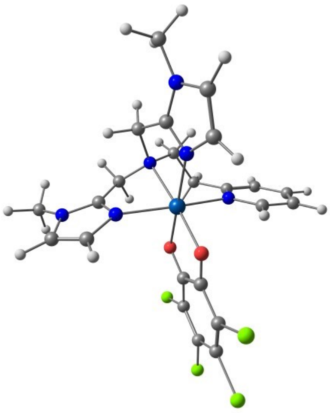

[Co(BMIMAPY)TCSQ]$^+$

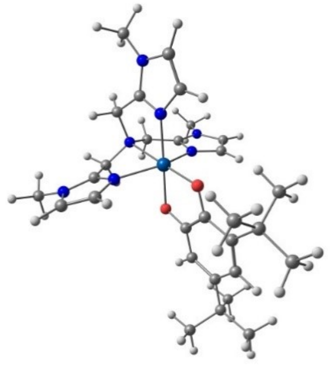

$[Co(TMIMA)DTBSQ]^+$

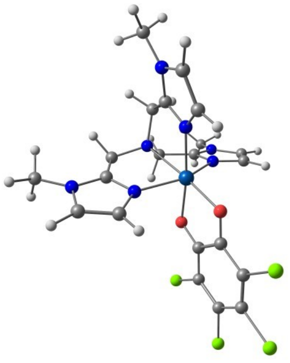

$[Co(TMIMA)TCSQ]^+$

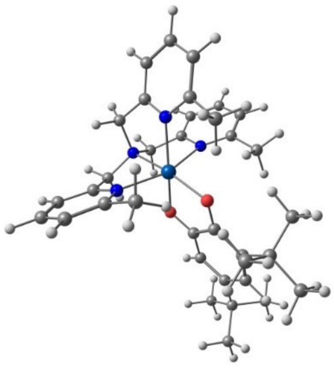

$[Co(Me_3TPA)DTBSQ]^+$

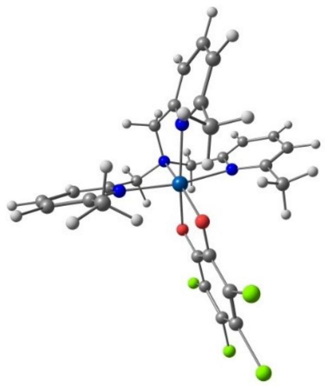

$[Co(Me_3TPA)TCSQ]^+$

Figure S7: DFT optimized structures of the different Co complexes discussed in the main text.

Table S6: DFT calculated structure energies of the Co(III)Cat singlet state and Co(II)SQ quintet state for the different Co complexes.

	**E (kJ mol^{-1})**	**E_{rel} (kJ mol^{-1})**
**[Co($PzPy_2$)(DTBCat)]$^+$**	-8012771.22	0.00
**[Co($PzPy_2$)(DTBSQ)]$^+$**	-8012754.26	16.96
**[Co(BMIMAPY)(DTBCat)]$^+$**	-8058091.48	0.00
**[Co(BMIMAPY)(DTBSQ)]$^+$**	-8058082.11	9.37
**[Co(TMIMA)(DTBCat)]$^+$**	-8000085.38	0.00
**[Co(TMIMA)(DTBSQ)]$^+$**	-8000086.44	-1.06
**[Co(Me_3TPA)(DTBCat)]$^+$**	-8174033.44	0.00
**[Co(Me_3TPA)(DTBSQ)]$^+$**	-8174072.58	-39.14

**[Co($PzPy_2$)(TCCat)]$^+$**	-12013591.25	0.00
**[Co($PzPy_2$)(TCSQ)]$^+$**	-12013554.84	36.41
**[Co(BMIMAPY)(TCCat)]$^+$**	-12058918.05	0.00
**[Co(BMIMAPY)(TCSQ)]$^+$**	-12058889.40	28.65
**[Co(TMIMA)(TCCat)]$^+$**	-12000912.82	0.00
**[Co(TMIMA)(TCSQ)]$^+$**	-12000894.91	17.91
**[Co(Me_3TPA)(TCCat)]$^+$**	-12174872.03	0.00
**[Co(Me_3TPA)(TCSQ)]$^+$**	-12174873.92	-1.89

Table S7: First coordination sphere bond lengths from DFT calculations.

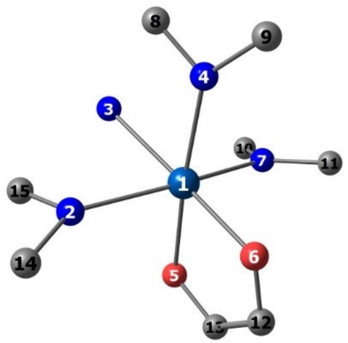

Scheme: atom numbers of first coordination sphere (simplified structure).

	**Co – N2**	**Co – N3**	**Co – N4**	**Co – N7**	**Co – O5**	**Co – O6**	**C13 – O5**	**C12 – O6**	**C12 – C13**
**$[Co(PzPy_2)(DTBCat)]^+$**	1.917	1.982	1.929	1.928	1.853	1.846	1.362	1.366	1.403
**$[Co(PzPy_2)(DTBSQ)]^+$**	2.156	2.246	2.098	2.102	2.044	1.980	1.287	1.302	1.463
**$[Co(BMIMAPY)(DTBCat)]^+$**	1.919	2.060	1.928	1.956	1.862	1.840	1.359	1.364	1.404
**$[Co(BMIMAPY)(DTBSQ)]^+$**	2.121	2.295	2.088	2.170	2.053	1.997	1.291	1.293	1.464
**$[Co(TMIMA)(DTBCat)]^+$**	1.914	2.043	1.919	1.909	1.857	1.832	1.360	1.364	1.405
**$[Co(TMIMA)(DTBSQ)]^+$**	2.113	2.401	2.076	2.103	2.062	1.979	1.287	1.296	1.463
**$[Co(Me_3TPA)(DTBCat)]^+$**	2.018	1.971	2.063	2.006	1.842	1.885	1.353	1.364	1.399
**$[Co(Me_3TPA)(DTBSQ)]^+$**	2.177	2.165	2.156	2.340	2.037	2.012	1.288	1.298	1.460

**[Co(PzPy$_2$)(TCCat)]$^+$**	1.922	1.973	1.920	1.924	1.865	1.852	1.343	1.349	1.403
**[Co(PzPy$_2$)(TCSQ)]$^+$**	2.150	2.224	2.089	2.094	2.067	1.999	1.275	1.286	1.460
**[Co(BMIMAPY)(TCCat)]$^+$**	1.916	2.047	1.919	1.954	1.870	1.852	1.341	1.345	1.404
**[Co(BMIMAPY)(TCSQ)]$^+$**	2.113	2.279	2.076	2.163	2.079	2.016	1.276	1.280	1.462
**[Co(TMIMA)(TCCat)]$^+$**	1.911	2.030	1.909	1.906	1.864	1.845	1.342	1.345	1.406
**[Co(TMIMA)(TCSQ)]$^+$**	2.098	2.383	2.071	2.102	2.083	1.996	1.274	1.282	1.461
**[Co(Me$_3$TPA)(TCCat)]$^+$**	1.977	1.957	2.040	2.003	1.861	1.859	1.340	1.339	1.400
**[Co(Me$_3$TPA)(TCSQ)]$^+$**	2.156	2.140	2.147	2.289	2.077	2.011	1.274	1.284	1.457

Table S8: Variation of bond lengths of the different Co complexes upon transition from ls-Co(III)Cat to hs-Co(II)SQ redox isomers.

	**Δ(Co – N2)**	**Δ(Co – N3)**	**Δ(Co – N4)**	**Δ(Co – N7)**	**Δ(Co – O5)**	**Δ(Co – O6)**	**Δ(C13 – O5)**	**Δ(C12 – O6)**	**Δ(C12 – C13)**
**[Co(PzPy$_2$)(DTBCat)]$^+$**	0.24	0.26	0.17	0.17	0.19	0.13	-0.08	-0.06	0.06
**[Co(BMIMAPY)(DTBCat)]$^+$**	0.20	0.24	0.16	0.21	0.19	0.16	-0.07	-0.07	0.06
**[Co(TMIMA)(DTBSQ)]$^+$**	0.20	0.36	0.16	0.19	0.21	0.15	-0.08	-0.09	0.05
**[Co(Me$_3$TPA)(DTBSQ)]$^+$**	0.16	0.19	0.09	0.33	0.19	0.13	-0.06	-0.07	0.06

	Δ(Co – N2)	Δ(Co – N3)	Δ(Co – N4)	Δ(Co – N7)	Δ(Co – O5)	Δ(Co – O6)	Δ(C13 – O5)	Δ(C12 – O6)	Δ(C12 – C13)
**[Co(PzPy$_2$)(TCCat)]$^+$**	0.23	0.25	0.17	0.17	0.20	0.15	-0.07	-0.06	0.06
**[Co(BMIMAPY)(TCCat)]$^+$**	0.20	0.23	0.16	0.21	0.21	0.16	-0.07	-0.07	0.06
**[Co(TMIMA)(TCCat)]$^+$**	0.19	0.35	0.16	0.20	0.22	0.15	-0.07	-0.06	0.06
**[Co(Me$_3$TPA)(TCSQ)]$^+$**	0.18	0.18	0.11	0.29	0.22	0.15	-0.07	-0.06	0.06

*$\Delta = bond_{Co(II)SQ} - bond_{Co(III)Cat}$

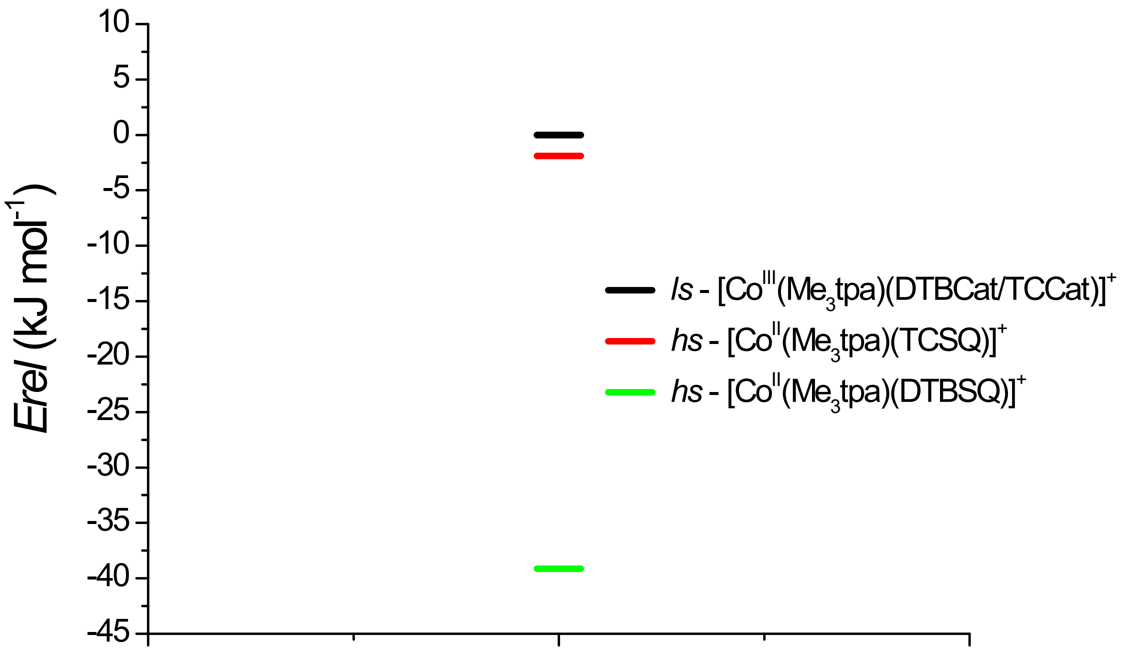

Figure S8: Calculated relative energy for the [Co(Me$_3$tpa)(DTBCat)]$^+$ and [Co(Me$_3$tpa)(TCCat)]$^+$ complexes.

References

1) G. A. Bain and J. F. Berry, *J. Chem. Educ.*, 2008, **85**, 532-536.

2a) Bruker. SAINT v8.34A. Bruker AXS Inc., 2013, Madison, Wisconsin, USA; 2b) Bruker APEX2 v2014.5-0. Bruker AXS Inc., 2007, Madison, Wisconsin, USA.

3) G. M. Sheldrick, *Acta Cryst.*, 2008, **A64**, 112-122.

4) L. J. Farrugia, *J. Appl. Cryst.* 2012, **45**, 849-854.

5) Bruker *SADABS,* Bruker AXS Inc., 2014, Madison, Wisconsin, USA.

6) C. K. Johnson, C. K. Crystallographic Computing, F. R. Ahmed, 1970, Copenhagen,

Munksgaard.

7) C. F. Macrae, P. R. Edgington, P. McCabe, E. Pidcock, G. P. Shields, R. Taylor, M. Towler and J. Van de Streek, *J. Appl. Crystallogr.*, 2006, **39**, 453−457.

8) G. K. Gransbury, M.-E. Boulon, S. Petrie, R. W. Gable, R. J. Mulder, L. Sorace, R. Stranger and C. Boskovic, *Inorg. Chem*., 2019, **58**, 4230-4243.

9) F. Neese, *WIREs Comput Mol Sci*, 2012, **2**, 73–78.

10) F. Neese, *WIREs Comput Mol Sci*, 2018, **8**, e1327.

11) F. Neese, F. Wennmohs, U. Becker and C. Riplinger, *J. Chem. Phys.*, 2020, **152**, 224108.
